# Changes in Choroidal Thickness and Its Effects on the Refractive Outcome After Surgical Treatment of Cataract Using Phacoemulsification Combined With Goniosynechialysis in Patients With Primary Angle Closure/Glaucoma

**DOI:** 10.1155/joph/7173240

**Published:** 2025-12-12

**Authors:** Siqi Guo, Hao You, Zhen Jiang, Kaihui Chen, Ruifeng Li, Ling Yu

**Affiliations:** ^1^ Department of Ophthalmology, Army Medical Center of PLA, Army Medical University, Chongqing, 400042, China, tmmu.edu.cn

**Keywords:** changes and effects, choroidal thickness, PAC/PACG, Phaco-GSL

## Abstract

**Purpose:**

To evaluate changes in choroidal thickness (CT) and their association with refractive outcomes after phacoemulsification combined with goniosynechialysis (Phaco‐GSL) in patients with primary angle‐closure/glaucoma (PAC/PACG).

**Methods:**

This study involved 79 eyes from 54 patients with PAC/PACG who underwent Phaco‐GSL. Intraocular pressure (IOP), best‐corrected visual acuity (BCVA), and CT were measured preoperatively and at 1 week (W1), 1 month (M1), 3 months (M3), and 6 months (M6) postoperatively. In addition, anterior chamber depth (ACD), axial length (AL), visual field mean deviation (MD), and mean refractive error (MRE) were recorded preoperatively and at the final follow‐up visit. Correlations between ocular biometric parameters and MRE were analyzed using appropriate statistical methods.

**Results:**

Within 6 months after Phaco‐GSL, CT measured at 13 predefined locations showed an initial postoperative increase, followed by a gradual decline, eventually returning to baseline levels. IOP decreased at W1 and stabilized at M6. In addition, subfoveal choroidal thickness (SFCT) was negatively correlated with IOP preoperatively (*p* < 0.05) and AL both preoperatively and at M6 (*p* < 0.01) but positively related to MD at M6. Moreover, CT changes were positively correlated with MRE (*p* < 0.05), while changes in ACD, AL, and IOP were not associated with MRE (all, *p* > 0.05).

**Conclusion:**

These findings suggest that CT initially increases and then decreases over a 6‐month period following Phaco‐GSL. CT is associated with IOP, AL, and MD reductions. Furthermore, CT changes are associated with an increase in MRE, offering valuable insights into the refractive shifts in PAC/PACG patients after Phaco‐GSL.

## 1. Introduction

Primary angle‐closure (PAC) and primary angle‐closure glaucoma (PACG) are subtypes of glaucoma, with particularly high prevalence in Asia, especially in China [1, 2]. The precise mechanism underlying PAC has not been fully elucidated. Some scholars propose that the expansion of the choroid plays an important role in angle closure. Specifically, choroidal thickening may elevate posterior segment pressure, creating a transvitreal pressure gradient that pushes the lens–iris diaphragm anteriorly, resulting in anterior chamber shallowing and narrowing of the iridocorneal angle [3]. Additionally, healthy individuals with shallow anterior chambers or short axial length (AL) are susceptible to acute angle‐closure attacks [4]. Therefore, the exact mechanism for the onset of PAC/PACG deserves further attention.

Studies have shown that after glaucoma surgery, a decrease in intraocular pressure (IOP) may lead to an increase in choroidal blood volume, contributing to a decrease in AL and an increased choroidal thickness (CT) [5]. However, the exact role of CT in glaucoma remains undetermined. In recent years, phacoemulsification combined with intraocular lens (IOL) implantation and goniosynechialysis (Phaco‐IOL‐GSL) has become a prominent therapeutic option for PAC/PACG due to its safety and efficacy [6]. Our previous study showed that patients with PAC/PACG often experience a myopic shift after phacoemulsification combined with goniosynechialysis (Phaco‐GSL) [7]. However, it remains unclear whether there is a correlation between IOP and CT pre‐ and postoperatively in patients who undergo Phaco‐GSL. In addition, it remains to be understood whether CT changes contribute to refractive error after surgery.

In this study, we used the enhanced depth imaging optical coherence tomography (EDI‐OCT) technique, as described by Spaide et al. to perform multipoint measurements and multiangle analyses [8]. EDI‐OCT enables the acquisition of high‐quality images of the choroidal membrane, which is conducive to the observation of CT [9–12]. On the basis of these measurements and analyses, we explored the characteristics and influencing factors of CT pre‐ and postoperatively in patients with PAC/PACG. We also analyzed the effect of CT on the refractive outcome of patients with PAC/PACG.

A growing body of research has demonstrated the impact of CT on refractive outcomes following intraocular surgery [13, 14]. For instance, Song et al. emphasized the importance of preoperative subfoveal choroidal thickness (SFCT) and the variations between pre‐ and postoperative measurements in predicting refractive errors in patients with PAC undergoing cataract surgery [13]. Furthermore, studies have revealed a connection between AL and refractive outcomes, indicating that a longer AL is associated with higher refractive errors postcataract surgery [15, 16]. Patients with PACG often experience poor refractive precision due to their shorter AL and shallower anterior chambers [17].

Therefore, the current study aimed to systematically evaluate changes in CT, AL, anterior chamber depth (ACD), and other anterior segment parameters in patients with PAC/PACG who underwent Phaco‐GSL. We further sought to determine whether variations in CT and other ocular biometric parameters contribute to refractive outcomes following surgery.

## 2. Materials and Methods

### 2.1. Patients

We conducted a prospective interventional study on Chinese subjects, which was in accordance with the principles of the Declaration of Helsinki. This study was approved by the Ethics Committee of the Characteristic Medical Center of the PLA Army (No. 2022‐133). Written informed consent was obtained from all the subjects. We recruited PAC/PACG patients who received Phaco‐GSL at the Chongqing Army Medical Center’s Glaucoma Clinic between June 2022 and December 2022. Inclusion criteria included (1) diagnosis of PAC/PACG with cataract and attendance at our hospital for Phaco‐GSL; (2) age ≥ 45 years and willingness to provide written informed consent for participation and data collection; (3) availability of high‐quality macular scans obtained via EDI‐OCT and accurate measurements of CT; and (4) absence of any preoperative ocular pathology other than PAC/PACG and cataract. Exclusion criteria were as follows: (1) presence of refractive media opacity or low‐quality on OCT that interfered with accurate measurement of CT; (2) presence of choroidal pathology, including choroidal neovascularization, melanoma, or choroidal atrophy; (3) presence of high myopia (spherical equivalent [SE] < −6.0 diopters [D]) or hyperopia (refractive > +6.0D); and (4) presence of systemic diseases that affect ocular status or interfere with surgery and examination, such as neurodegenerative diseases and rheumatic immunological diseases.

### 2.2. Ophthalmologic Examination

An experienced ophthalmologist who was blinded to the clinical diagnosis of each patient performed comprehensive ophthalmologic examinations, such as slit‐lamp biomicroscopy and fundus examination. Data on IOP, manifest refraction, best‐corrected visual acuity (BCVA), AL, ACD, CT, and visual field (VF) index (VFI) mean deviation (MD) were collected before surgery. IOP, CT, and BCVA were reassessed on postoperative Day 1 and at follow‐up visits at 1 week (W1), 1 month (M1), 3 months (M3), and 6 months (M6). Manifest refraction, ACD, AL, and MD were reevaluated at the final follow‐up visit. Mean refractive error (MRE) was calculated at M6 as the difference between the predicted and actual postoperative SE values.

**Figure 1:**
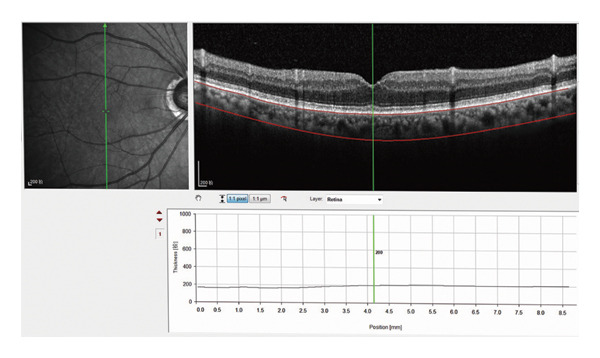
Measurement of choroidal thickness.

AL and ACD were measured using the IOL‐Master 700 (Carl Zeiss Meditec, Jena, Germany), and the online Barrett Universal II formula was used for IOL calculations. Choroidal imaging was conducted using a Spectralis HRA OCT (Heidelberg Engineering, Heidelberg, Germany) in EDI mode, which automatically reverses the inverted image for direct EDI‐OCT acquisition. The imaging protocol consisted of 13 horizontal B‐scans, each comprising 768 A‐scans, within a scan area of 530 rectangles covering the macula and optic nerve head. Each B‐scan was averaged from 100 frames using active eye tracking. The scan line intersecting the foveal center, confirmed manually using horizontal and vertical reference lines, was selected for CT analysis. SFCT was defined as the vertical distance from the outer surface of the hyperreflective retinal pigment epithelium (RPE) to the inner scleral border. CT was manually measured at 13 predefined locations (the subfoveal region, and at 500, 1000, and 3000 μm nasally, temporally, superiorly, and inferiorly from the fovea) (Figure 1). All patients underwent a standard automated VF (SAP) examination preoperatively and at M6, and they were tested using the Swedish Interactive Threshold algorithm, Fast 24‐2, on the Humphrey VF analyzer 750II (Carl Zeiss Meditec, Dublin, CA). If any abnormal results were identified, VF tests were repeated. Only reliable VF results (fixation losses < 20% and false‐positive and false‐negative error rates < 10%) were included in the final analysis. All VF results were independently reviewed by two ophthalmologists. MD values were obtained using the Humphrey Field Analyzer. In addition, an experienced eye technician who was blinded to the subjects’ clinical diagnoses performed an optic disc OCT using Zeiss CirrusTM HD‐OCT (5000–8042) and measured the retinal nerve fiber layer (RNFL)‐to‐cup‐disc ratio using the Optic Disc Cube 200,200 mode. In addition, ultrasound biomicroscopy (Tianjin Suowei Electronic Technology Co., Ltd.) and gonioscopy were used to diagnose angle‐closure.

### 2.3. Surgical Procedures

We performed Phaco‐GSL surgeries using our previously published protocol [7]. All surgeries were conducted by a single experienced surgeon (Ling Yu) under topical (surface) anesthesia with oxybuprocaine hydrochloride at a dose of 2 drops per eye. After Phaco‐IOL, angle separation was performed under a gonioscopic view. Postoperative treatment included the administration of dexamethasone–tobramycin eye drops, levofloxacin eye drops, and polyethylene glycol eye drops, each applied four times daily for 2 weeks.

### 2.4. Statistical Analysis

Data were presented as either the median and interquartile range or mean ± standard deviation (SD). The D’Agostino–Pearson test was used to assess the normality of variable distributions. Repeated‐measures analysis of variance (rmANOVA) was used to evaluate dynamic variations in CT measured at each measurement point across time. Nonparametric tests were used to analyze the dynamic changes in IOP values from the preoperative baseline to M6. Correlations between CT and ocular biometric parameters were examined using Pearson’s correlation analysis for normally distributed data and Spearman’s correlation analysis for nonnormally distributed data. A *p* value of less than 0.05 was considered statistically significant.

## 3. Results

A total of 58 patients (85 eyes) were initially recruited from our outpatient clinic, but six eyes were excluded due to poor image quality. Finally, 79 eyes from 54 patients (mean age, 63.30 ± 6.76 years; age range, 46–76 years) with PAC/PACG were included. Among them, there were 15 male patients and 39 female patients. The median preoperative values of the measured parameters were as follows: AL, 22.62 mm (IQR: 22.12–22.96 mm); ACD, 2.32 mm (IQR: 2.17–2.53 mm); predicted SE, −0.39 D (IQR: −0.46 to 0.25 D); actual SE, 0.50 D (IQR: −0.50 to 1.38 D); IOP, 16.70 mmHg (IQR: 14.10–23.20 mmHg); BCVA, 0.10 log MAR (IQR: 0.10–0.22); and MD, −6.47 dB (IQR: −14.85 to 3.16 dB). Baseline demographic and clinical characteristics are summarized in Table 1.

**Table 1:** Basic characteristics of the patients.

Parameter	Values
Eyes (*n*)	79
Mean age (years)	63.30 ± 6.76
Male/female (*n*)	15/39
Preop AL (mm)	22.62 (22.12, 22.96)
Postop (6m) AL(mm)^a^	22.54 (21.98, 22.83)
Preop ACD (mm)	2.32 (2.17, 2.53)
Postop (6m) ACD (mm)^b^	3.97 (3.68, 4.16)
Predicted SE (D)	−0.39 (−0.46, −0.25)
SE (D)	0.50 (−0.50, 1.38)
IOP (mmHg)	16.70 (14.10, 23.20)
BCVA	0.10 (0.10, 0.22)
Preop MD	−6.47 (−14.85, −3.16)
Postop (6m) MD^c^	−4.25 (−15.00, −1.64)

*Note:* Values represent mean ± SD or median and interquartile range.

Abbreviations: ACD, anterior chamber depth; AL, axial length; BCVA, best‐corrected visual acuity; D, diopter; IOP, intraocular pressure; MD, mean deviation; SE, spherical equivalent.

^a^Postop (6m) AL was less than preop AL, *p* < 0.001.

^b^Postop (6m) ACD was greater than preop ACD, *p* < 0.001.

^c^Postop (6m) MD was greater than preop MD, *p* < 0.001.

**Figure 2:**
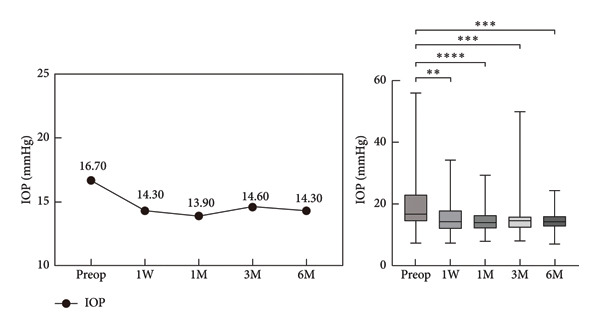
Comparison of intraocular pressure (IOP) preoperative and postoperative, ^∗^ *p* < 0.05; ^∗∗^ *p* < 0.01; ^∗∗∗^ *p* < 0.001; ^∗∗∗∗^ *p* < 0.0001.

The median preoperative IOP was 16.70 mmHg (IQR: 14.10–23.20 mmHg). Postoperatively, it decreased significantly at W1 (14.30 mmHg [IQR: 11.90–18.00 mmHg]), M1 (13.90 mmHg [IQR: 12.10–16.50 mmHg]), M3 (14.60 mmHg [IQR: 12.20–16.10 mmHg]), and M6 (14.30 mmHg [IQR: 12.60–16.20 mmHg]). There were no statistically significant differences in IOP values between different postoperative time points (Figure 2).

**Figure 3:**
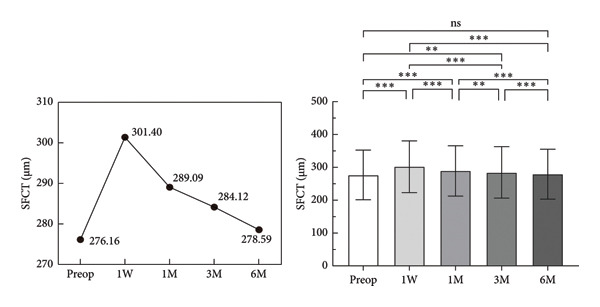
Comparison of subfoveal choroidal thickness (SFCT) preoperative and postoperative, ^∗^ *p* < 0.05; ^∗∗^ *p* < 0.01; ^∗∗∗^ *p* < 0.001; ns: no significance.

The mean preoperative SFCT was 276.16 ± 75.73 mm. Postoperatively, it increased significantly at W1 (*p* < 0.001), M1 (*p* < 0.001), and M3 (*p* < 0.05). The SFCT value increased to a maximum at W1 and then gradually decreased, and it returned to the preoperative level at M6 (Figure 3, Table 2). In addition, we compared the CT values of the remaining 12 points between the preoperative and postoperative time points. We found that the nasal CT 1 (NCT1) and temporal CT 1 (TCT1) increased significantly at W1, M1, and M3. The superior CT 1 (SCT1), SCT2, and SCT3; the inferior CT 1 (ICT1) and ICT2; NCT2, and TCT2 increased significantly at W1 and M1 (all, *p* < 0.05). In addition, ICT3, NCT3, and TCT3 increased significantly at W1 (Supporting Files 1–4).

**Table 2:** Comparison of subfoveal choroidal thickness at different stages.

Parameter	Mean ± SD (μm)	*F* value	*p* value	Post hoc
*Subfoveal choroidal thickness*		28.93	< 0.001	*P*_1−2_ < 0.001 *P*_1–3_ < 0.001 *P*_1–4_ < 0.05 *P*_1–5_ > 0.05 *P*_2−3_ < 0.001 *P*_2–4_ < 0.001 *P*_2–5_ < 0.001 *P*_3−4_ < 0.05 *P*_3–5_ < 0.001 *P*_4−5_ < 0.05
Preop	276.16 ± 75.73			
First week postop	301.40 ± 78.66			
First month postop	289.09 ± 76.57			
Third month postop	284.12 ± 78.16			
Sixth month postop	278.59 ± 76.07			

*Note:* *p* < 0.05 was considered statistically significant.

Abbreviation: SD, standard deviation.

We analyzed the correlation of SFCT with IOP, AL, ACD, and MD pre‐ and postoperatively. SFCT showed no correlation with IOP at W1, M1, M3, or M6 (Table 3). There was a weak negative correlation between preoperative SFCT and IOP. Additionally, SFCT was negatively correlated with AL pre‐ and postoperatively, and it was significantly positively associated with MD at M6. No significant correlation was observed between ACD and SFCT (Table 4).

**Table 3:** Correlation between intraocular pressure and subfoveal choroidal thickness.

Intraocular pressure	Values (mmHg)	Correlation *r*	*p* value
Preop	16.70 (14.10, 23.20)	−0.267736	0.017057
First week postop	14.30 (11.90, 18.00)	0.134370	0.237749
First month postop	13.90 (12.10, 16.50)	0.020184	0.859854
Third month postop	14.60 (12.20, 16.10)	−0.122425	0.282444
Sixth month postop	14.30 (12.60, 16.20)	−0.007377	0.948551

*Note:* *p* < 0.05 was considered statistically significant.

**Table 4:** Correlation between subfoveal choroidal thickness and axial length, anterior chamber depth, and mean deviation.

Parameter	Values (mm)	Correlation *r*	*p* value
*Axial length*			
Preop	22.62 (22.12, 22.96)	−0.339742	0.002190
Postop (6m)	22.54 (21.98, 22.83)	−0.305743	0.006141

*Anterior chamber depth*			
Preop	2.32 (2.17, 2.53)	0.111401	0.328364
Postop (6m)	3.97 (3.68, 4.16)	−0.068571	0.548198

*Mean deviation*			
Preop	−6.47 (−14.85, −3.16)	0.218298	0.053270
Postop (6m)	−4.25 (−15.00, −1.64)	0.355310	0.001312

*Note:* *p* < 0.05 was considered statistically significant.

In addition, we analyzed the correlation of MRE with changes in ACD, AL, IOP, and CT at M6. We found a significant positive correlation between the degree of myopic drift and changes in CT. There were no other statistically significant correlations (Table 5).

**Table 5:** Correlation between MRE, changes in ACD, changes in AL, changes in IOP, and changes in choroidal thickness.

Parameter	Values	Correlation *r*	*p* value
Mean refractive error	0.25 ± 0.86	0.327698	0.003198
Changes in ACD	−1.59 (−1.81, −1.41)	0.015918	0.889263
Changes in AL	0.10 (0.07, 0.14)	−0.129994	0.253517
Changes in IOP	3.2 (−0.70, 9.20)	0.130249	0.252578

*Note:* *p* < 0.05 was considered statistically significant.

Abbreviations: ACD, anterior chamber depth; AL, axial length; IOP, intraocular pressure; MRE, mean refractive error.

## 4. Discussion

Building on our previous findings [7], this study aimed to characterize the dynamic changes in CT after Phaco‐GSL and to evaluate its associations with IOP, ACD, AL, MRE, and MD. We also sought to explore potential mechanisms underlying these changes.

The choroid is universally known to be a highly vascularized structure [18]. In addition, Kiyota et al. confirmed that choroidal blood flow is involved in the pathophysiology of glaucoma and that it is independently associated with glaucoma progression at a later stage [19]. We hypothesized that the sudden decrease in IOP may lead to choroidal hyperperfusion after Phaco‐GSL, resulting in vascular congestion and increased CT. This hypothesis aligns with previous studies by Kara et al. [20] and Quigley et al. [3]. Our results support this hypothesis: CT values at 13 different locations increased significantly in the early postoperative period, peaked at W1, and then decreased progressively to the baseline level by M6. Concurrently, the median IOP decreased significantly and remained stable throughout the follow‐up period. These findings suggest that the increase in CT is a self‐limiting, physiological response to changes in ocular perfusion pressure. Interestingly, we observed a weak negative correlation between preoperative SFCT and IOP, which disappeared postoperatively, implying that the dynamic CT changes after surgery are governed by additional regulatory mechanisms beyond baseline IOP‐CT relationships. In general, the association of changes in CT with IOP fluctuations in patients with PAC/PACG remains mysterious.

We also analyzed the correlations between CT and other ocular parameters like AL, ACD, and MD. Our findings revealed that CT was inversely correlated with AL both pre‐ and postoperatively and positively correlated with MD at M6 postoperatively. No correlation was observed between CT and ACD. These findings are consistent with previous reports [9, 21, 22]. The inverse relationship between CT and AL is well established. For example, studies have shown that during accommodation, AL increased while CT decreased at both central and peripheral positions [23]. Another research revealed that IOP‐related choroidal thinning could cause axial elongation [24], a finding further supported by recent work from Hata et al. [25]. Moreover, a positive correlation between CT and MD has been reported. For instance, Moghimi et al. [26] found that worse MD was the only factor that was associated with a thinner choroid when the IOP was high. In contrast, both our current study and that of Singh et al. [27] revealed no correlation between MD and CT preoperatively. Notably, we observed postoperative improvement in MD after Phaco‐GSL, consistent with previous findings that cataract extraction with monofocal IOL implantation can lead to functional VF improvement in eyes with mild to moderate glaucoma [28–30]. These results add to the ongoing debate regarding the potential of CT as a biomarker for glaucoma severity. A previous study reported that thicker choroids are associated with a higher IOP and a more severe disease in patients with PACG, proposing that intereye asymmetry of CT can be used as a disease severity‐predictor [27]. In contrast, other studies have shown that while elevated macular CT may be a prevalent anatomical feature of primary angle‐closure disease (PACD) eyes, macular CT is not a useful diagnostic tool for determining the severity of POAG or PACG [31, 32]. Our finding of a positive correlation between CT and MD at M6 adds a novel dimension to this ongoing discussion, suggesting that the relationship may be dynamic and change in the postoperative period.

In the present study, a significant positive correlation was observed between the degree of refractive error and the increase in CT at M6. However, changes in ACD, AL, and IOP showed no such association. As far as we are aware, no other published work has assessed the relationship between MRE and the modifications in CT following Phaco‐GSL. In our previous study [7], we reported that eyes with shorter AL and shallower preoperative anterior chambers were more likely to exhibit postoperative ACD deepening, which contributed to a myopic shift after cataract surgery [33]. Furthermore, the evaluation of CT changes might help progress our understanding of a refractive shift in patients with PAC/PACG. Our findings are consistent with Song et al. [13], who found that in patients with PAC, preoperative SFCT and the distinction between pre‐ and post‐operative SFCT were important predictors of refractive error after cataract surgery. They proposed that a large myopic shift at the postoperative refractive test was linked to higher CT before cataract surgery.

The exact mechanism triggering acute attacks in PACG eyes remains unknown. One proposed mechanism is the potential relationship between anterior CT (ACT) and IOP, as well as the narrowed anterior chamber in patients with PACD. Fei Li et al. reported that the Valsalva maneuver thickened the anterior choroid, which resulted in a constricted anterior chamber [34]. The choroidal dilation theory suggests that increased IOP in PAC/PACG eyes may significantly influence posterior CT (PCT) [24, 25]. These results indicate that anterior choroidal parameters may be more closely associated with etiology and progression [35], consistent with our observation that IOP is independent of PCT. Secondly, inflammatory mechanisms may play a role. Previous studies have shown that the aqueous humor of eyes with acute PACG had markedly higher levels of inflammation‐related cytokines, including interleukin‐6 (IL‐6), IL‐8, tumor necrosis factor alpha (TNF‐α), and monocyte chemotactic protein (MCP) [36–38]. Some scholars hold that this cytokine elevation triggers sudden attacks [39]. Few studies have explored the relationship between CT and cytokine levels. Zeng et al. found an inverse correlation between the neutrophil‐to‐lymphocyte ratio (NLR) and SFCT with peripheral blood IL‐6 levels in acute PACG patients [40], suggesting that changes in CT may be associated with postoperative inflammation. In our study, the mean CT increased significantly at W1 but started decreasing at M1 and M3, then returned to the preoperative level at M6. These trends align with findings from Ibrahim [41]. Although the exact mechanism by which cataract surgery induces retinal and choroidal inflammation is not fully understood, supporting evidence exists. Proinflammatory gene expression and protein release were observed in the retina and choroid following cataract surgery in a rat model [42]. These findings support the hypothesis that inflammatory changes observed in the posterior segment may be a secondary response to the inflammatory processes initiated in the anterior segment. Collectively, the evidence highlights the importance of future research focused on elucidating the mechanisms underlying CT changes after Phaco‐GSL, especially the contribution of intraocular inflammatory processes.

Studies have shown that factors such as age [10, 35, 43, 44], diastolic blood pressure [45], AL [27], refraction [27], diurnal variation [25], use of antiglaucoma drugs [20], and other factors are associated with CT changes. However, our study employed a within‐subject, before‐and‐after design, allowing each participant to serve as their own control. This methodological approach effectively minimized the influence of interindividual variability and mitigated the impact of these confounding factors. Furthermore, in our study, baseline and follow‐up data were collected at the same clinic time, thereby reducing the potential bias introduced by diurnal variations in CT. Nonetheless, several limitations should be acknowledged when interpreting our findings. First, the study is constrained by a relatively small cohort, which may restrict the generalizability of the findings to the broader population. Second, the follow‐up period of this study was relatively short, precluding the assessment of long‐term trends and dynamic changes over time. Third, the potential influence of eye drops on CT was not accounted for, introducing measurement variability. Finally, manually performed CT measurements are inherently susceptible to examiner bias, which may have affected the accuracy of the data. To strengthen the validity and applicability of these findings, future studies should incorporate larger cohorts, long‐term follow‐up periods, and standardized measurement techniques.

## 5. Conclusion

The current study showed that CT at all 13 measured locations increased significantly after Phaco‐GSL in the early postoperative period and then decreased to near‐baseline values within 6 months. A negative correlation was observed between SFCT and IOP preoperatively and between SFCT and AL pre‐ and postoperatively. Additionally, a positive correlation was noted between SFCT and MD at M6. Importantly, changes in CT showed a positive correlation with MRE. These observations suggest that the change in CT after Phaco‐GSL may serve as a useful adjunctive parameter in predicting refractive shifts following the surgery. Further research involving larger cohorts, extended follow‐up periods, and mechanistic investigations is warranted to better elucidate the factors contributing to choroidal remodeling and its clinical implications.

## Ethics Statement

This study was approved by the Ethics Committee of the Characteristic Medical Center of the PLA Army (No. 2022‐133). This study was performed in line with the principles of the Declaration of Helsinki.

## Consent

Written informed consent was obtained from all individual participants for the publication of their anonymized data and any potentially identifiable images presented in this study.

## Disclosure

All authors have read and approved the manuscript in its current state.

## Conflicts of Interest

The authors declare no conflicts of interest.

## Author Contributions

Conception and design of study: L.Y., R.L.; collection, analysis, and interpretation of data: Z.J., K.C., H.Y., S.G.; writing of the article S.G., H.Y.; critical revision of the article: L.Y.; patients referring: Z.J., H.Y.

S.G. and H.Y. contributed equally to this work.

## Funding

This work was supported by the National Natural Science Foundation of China (No. 82070962).

## Supporting Information

Additional supporting information can be found online in the Supporting Information section.

The following Supporting Information is available with this manuscript:

## Supporting information


**Supporting Information 1** Supporting File 1: Comparison of superior choroidal thickness at different stages.

**Supporting Information 2** Supporting File 2: Comparison of inferior choroidal thickness at different stages.

**Supporting Information 3** Supporting File 3: Comparison of Nasal choroidal thickness at different stages.

**Supporting Information 4** Supporting File 4: Comparison of temporal choroidal thickness at different stages.

**Supporting Information 5** Supporting File 5: Figure depicting comparisons of choroidal thickness in different quadrants at different stages.

## Data Availability

The data that support the findings of this study are available on request from the corresponding author. The data are not publicly available due to privacy or ethical restrictions.
