# Supplementary material for: Changes in Choroidal Thickness and Its Effects on the Refractive Outcome After Surgical Treatment of Cataract Using Phacoemulsification Combined With Goniosynechialysis in Patients With Primary Angle Closure/Glaucoma
Source: J Ophthalmol. 2025 Dec 12;2025:7173240. doi: 10.1155/joph/7173240 (PMC12767013; doi:10.1155/joph/7173240)
Supplement: Supplementary file 1 — Supporting Information 1 Supporting File 1: Comparison of superior choroidal thickness at different stages. [file JOPH-2025-7173240-s004.docx]

| **Supplemental file 1.** Comparison of superior choroidal thickness at different stages. | | | | |
| --- | --- | --- | --- | --- |
| **Parameter** | **Mean ± SD(μm)** | ***F* Value** | ***p* Value** | ***Post hoc*** |
| *Superior choroidal thickness 1* | | 24.03 | <0.001 | *p*1 < 0.001  *p*2 < 0.001  *p*3＞0.05  *p*4＞0.05  *p*5 < 0.001  *p*6 < 0.001  *p*7 < 0.001  *p*8 < 0.001  *p*9 < 0.001  *p*10＞0.05 |
| Pre-op | 279.40±73.36 |  |  |  |
| First week postop | 304.94±74.74 |  |  |  |
| First month postop | 293.27±74.06 |  |  |  |
| Third month postop | 284.38±76.95 |  |  |  |
| Sixth month postop | 280.98±72.93 |  |  |  |
| *Superior choroidal thickness 2* | | 16.76 | <0.001 | *p*1 < 0.001  *p*2 < 0.001  *p*3＞0.05  *p*4＞0.05  *p*5 < 0.05  *p*6 < 0.001  *p*7 < 0.001  *p*8 < 0.05  *p*9 < 0.001  *p*10＞0.05 |
| Pre-op | 275.37±70.88 |  |  |  |
| First week postop | 300.30±71.07 |  |  |  |
| First month postop | 290.42±71.99 |  |  |  |
| Third month postop | 282.01±75.57 |  |  |  |
| Sixth month postop | 278.89±70.21 |  |  |  |
| *Superior choroidal thickness 3* | | 7.20 | <0.001 | *p*1 < 0.05  *p*2 < 0.05  *p*3＞0.05  *p*4＞0.05  *p*5＞0.05  *p*6＜0.05  *p*7 < 0.001  *p*8＜0.05  *p*9 < 0.001  *p*10＜0.05 |
| Pre-op | 255.90±62.28 |  |  |  |
| First week postop | 272.18±65.23 |  |  |  |
| First month postop | 268.43±63.97 |  |  |  |
| Third month postop | 262.23±64.01 |  |  |  |
| Sixth month postop | 254.53±58.07 |  |  |  |
