# Supplementary material for: Changes in Choroidal Thickness and Its Effects on the Refractive Outcome After Surgical Treatment of Cataract Using Phacoemulsification Combined With Goniosynechialysis in Patients With Primary Angle Closure/Glaucoma
Source: J Ophthalmol. 2025 Dec 12;2025:7173240. doi: 10.1155/joph/7173240 (PMC12767013; doi:10.1155/joph/7173240)
Supplement: Supplementary file 2 — Supporting Information 2 Supporting File 2: Comparison of inferior choroidal thickness at different stages. [file JOPH-2025-7173240-s005.docx]

| **Supplemental file 2.** Comparison of inferior choroidal thickness at different stages. | | | | |
| --- | --- | --- | --- | --- |
| **Parameter** | **Mean ± SD(μm)** | ***F* Value** | ***p* Value** | ***Post hoc*** |
| *Inferior choroidal thickness 1* | | 21.40 | <0.001 | *p*1 < 0.001  *p*2 < 0.05  *p*3＞0.05  *p*4＞0.05  *p*5 < 0.05  *p*6 < 0.001  *p*7 < 0.001  *p*8 < 0.05  *p*9 < 0.001  *p*10<0.001 |
| Pre-op | 273.38±74.80 |  |  |  |
| First week postop | 294.54±78.11 |  |  |  |
| First month postop | 284.62±76.94 |  |  |  |
| Third month postop | 278.58±77.65 |  |  |  |
| Sixth month postop | 272.00±75.07 |  |  |  |
| *Inferior choroidal thickness 2* | | 14.25 | <0.001 | *p*1 < 0.001  *p*2 < 0.05  *p*3＞0.05  *p*4＞0.05  *p*5 < 0.05  *p*6 < 0.001  *p*7 < 0.001  *p*8 < 0.05  *p*9 < 0.001  *p*10<0.05 |
| Pre-op | 263.95±71.58 |  |  |  |
| First week postop | 282.29±73.46 |  |  |  |
| First month postop | 273.75±76.67 |  |  |  |
| Third month postop | 267.99±72.35 |  |  |  |
| Sixth month postop | 262.49±71.06 |  |  |  |
| *Inferior choroidal thickness 3* | | 4.56 | <0.05 | *p*1 < 0.001  *p*2＞0.05  *p*3＞0.05  *p*4＞0.05  *p*5 < 0.05  *p*6 < 0.05  *p*7 < 0.05  *p*8＞0.05  *p*9＞0.05  *p*10<0.05 |
| Pre-op | 219.05±55.81 |  |  |  |
| First week postop | 231.51±58.42 |  |  |  |
| First month postop | 223.19±56.17 |  |  |  |
| Third month postop | 223.49±60.96 |  |  |  |
| Sixth month postop | 218.51±61.54 |  |  |  |
