# Supplementary material for: Changes in Choroidal Thickness and Its Effects on the Refractive Outcome After Surgical Treatment of Cataract Using Phacoemulsification Combined With Goniosynechialysis in Patients With Primary Angle Closure/Glaucoma
Source: J Ophthalmol. 2025 Dec 12;2025:7173240. doi: 10.1155/joph/7173240 (PMC12767013; doi:10.1155/joph/7173240)
Supplement: Supplementary file 3 — Supporting Information 3 Supporting File 3: Comparison of Nasal choroidal thickness at different stages. [file JOPH-2025-7173240-s002.docx]

| **Supplemental file 3.** Comparison of Nasal choroidal thickness at different stages. | | | | |
| --- | --- | --- | --- | --- |
| **Parameter** | **Mean ± SD(μm)** | ***F* Value** | ***p* Value** | ***Post hoc*** |
| *Nasal choroidal thickness 1* | | 14.67 | ＜0.001 | *p*1 < 0.001  *p*2＜0.05  *p*3＜0.05  *p*4＞0.05  *p*5 < 0.001  *p*6 < 0.001  *p*7 < 0.001  *p*8＞0.05  *p*9＜0.05  *p*10＞0.05 |
| Pre-op | 262.96±76.32 |  |  |  |
| First week postop | 285.59±80.69 |  |  |  |
| First month postop | 273.59±77.30 |  |  |  |
| Third month postop | 271.81±80.53 |  |  |  |
| Sixth month postop | 267.77±80.79 |  |  |  |
| *Nasal choroidal thickness 2* | | 9.43 | ＜0.001 | *p*1 < 0.001  *p*2＜0.05  *p*3＞0.05  *p*4＞0.05  *p*5 < 0.001  *p*6 < 0.05  *p*7 < 0.001  *p*8＞0.05  *p*9＞0.05  *p*10＞0.05 |
| Pre-op | 250.38±74.88 |  |  |  |
| First week postop | 268.94±78.63 |  |  |  |
| First month postop | 257.58±76.57 |  |  |  |
| Third month postop | 256.78±80.43 |  |  |  |
| Sixth month postop | 253.51±81.23 |  |  |  |
| *Nasal choroidal thickness 3* | | 8.57 | ＜0.001 | *p*1 < 0.05  *p*2＞0.05  *p*3＞0.05  *p*4＞0.05  *p*5＞0.05  *p*6 < 0.001  *p*7 < 0.001  *p*8＜0.001  *p*9＜0.001  *p*10＞0.05 |
| Pre-op | 175.46±67.48 |  |  |  |
| First week postop | 187.18±69.73 |  |  |  |
| First month postop | 182.44±69.66 |  |  |  |
| Third month postop | 171.19±70.35 |  |  |  |
| Sixth month postop | 166.52±67.35 |  |  |  |
