# Supplementary material for: Changes in Choroidal Thickness and Its Effects on the Refractive Outcome After Surgical Treatment of Cataract Using Phacoemulsification Combined With Goniosynechialysis in Patients With Primary Angle Closure/Glaucoma
Source: J Ophthalmol. 2025 Dec 12;2025:7173240. doi: 10.1155/joph/7173240 (PMC12767013; doi:10.1155/joph/7173240)
Supplement: Supplementary file 4 — Supporting Information 4 Supporting File 4: Comparison of temporal choroidal thickness at different stages. [file JOPH-2025-7173240-s001.docx]

| **Supplemental file 4.** Comparison of temporal choroidal thickness at different stages. | | | | | |
| --- | --- | --- | --- | --- | --- |
| **Parameter** |  | **Mean ± SD(μm)** | ***F* Value** | ***p* Value** | ***Post hoc*** |
| *Temporal choroidal thickness 1* | |  | 15.28 | ＜0.001 | *p*1 < 0.001  *p*2＜0.05  *p*3＜0.05  *p*4＞0.05  *p*5＜0.001  *p*6 < 0.001  *p*7 < 0.001  *p*8＞0.05  *p*9＜0.001  *p*10＜0.05 |
| Pre-op |  | 274.13±77.08 |  |  |  |
| First week postop |  | 296.96±77.62 |  |  |  |
| First month postop |  | 285.87±75.62 |  |  |  |
| Third month postop |  | 283.25±77.79 |  |  |  |
| Sixth month postop |  | 276.43±75.68 |  |  |  |
| *Temporal choroidal thickness 2* | |  | 9.56 | ＜0.001 | *p*1 < 0.001  *p*2＜0.05  *p*3＞0.05  *p*4＞0.05  *p*5＜0.05  *p*6 < 0.05  *p*7 < 0.001  *p*8＞0.05  *p*9＜0.001  *p*10＜0.05 |
| Pre-op |  | 271.62±76.74 |  |  |  |
| First week postop |  | 289.76±74.64 |  |  |  |
| First month postop |  | 281.30±74.30 |  |  |  |
| Third month postop |  | 278.66±76.56 |  |  |  |
| Sixth month postop |  | 269.68±74.14 |  |  |  |
| *Temporal choroidal thickness 3* | |  | 2.72 | ＜0.05 | *p*1 < 0.05  *p*2＞0.05  *p*3＞0.05  *p*4＞0.05  *p*5＞0.05  *p*6＞0.05  *p*7 < 0.05  *p*8＞0.05  *p*9＞0.05  *p*10＜0.05 |
| Pre-op |  | 238.10±65.98 |  |  |  |
| First week postop |  | 251.92±64.62 |  |  |  |
| First month postop |  | 245.61±59.53 |  |  |  |
| Third month postop |  | 246.11±60.05 |  |  |  |
| Sixth month postop |  | 239.38±55.28 |  |  |  |
