# Supplementary material for: Changes in Choroidal Thickness and Its Effects on the Refractive Outcome After Surgical Treatment of Cataract Using Phacoemulsification Combined With Goniosynechialysis in Patients With Primary Angle Closure/Glaucoma
Source: J Ophthalmol. 2025 Dec 12;2025:7173240. doi: 10.1155/joph/7173240 (PMC12767013; doi:10.1155/joph/7173240)
Supplement: Supplementary file 5 — Supporting Information 5 Supporting File 5: Figure depicting comparisons of choroidal thickness in different quadrants at different stages. [file JOPH-2025-7173240-s003.docx]

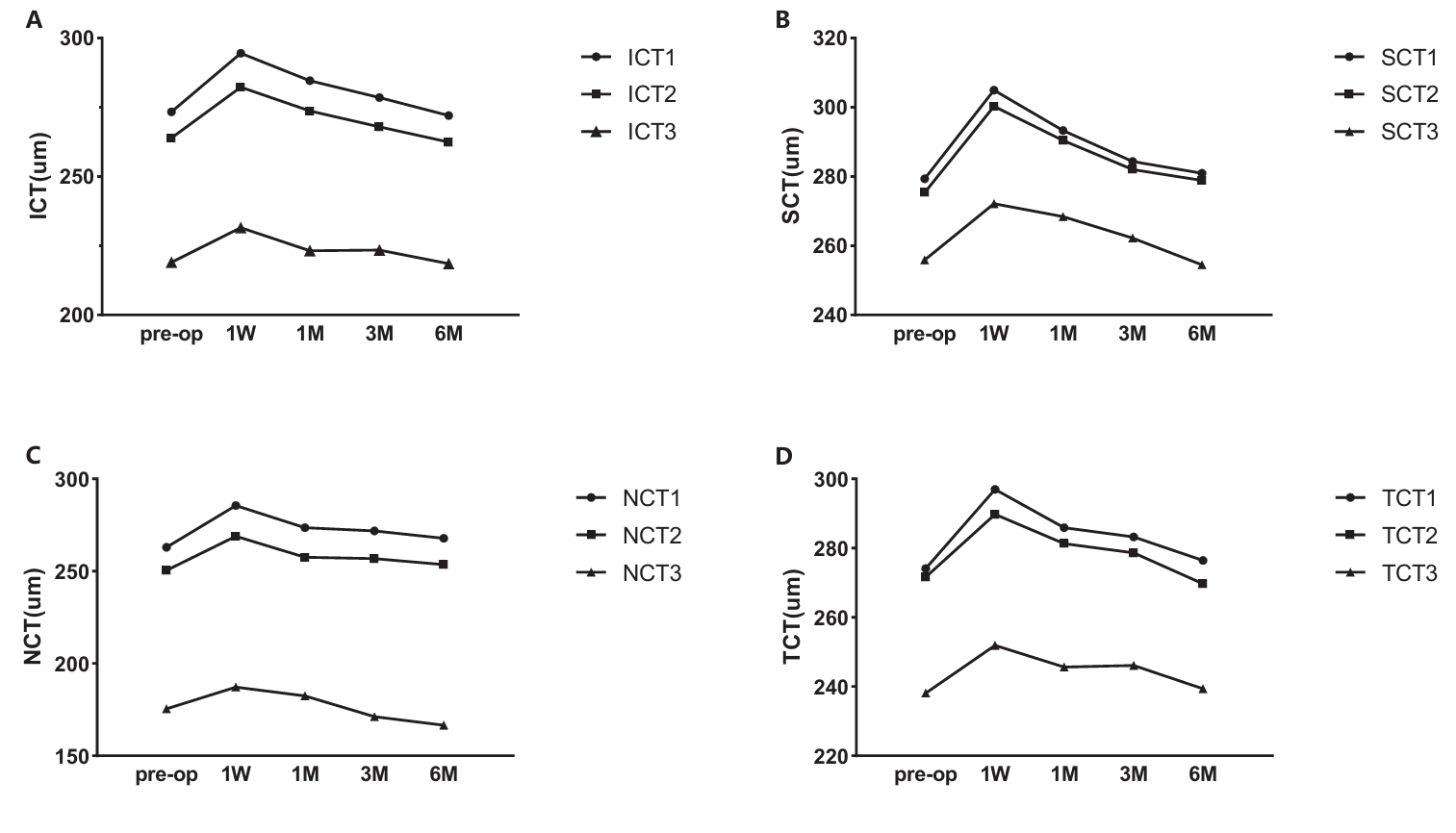


**Supplemental file 5**. A) Comparison of inferior choroidal thickness (ICT) at different stages. B) Comparison of superior choroidal thickness (SCT) at different stages. C) Comparison of Nasal choroidal thickness (NCT) at different stages. D) Comparison of temporal choroidal thickness (TCT) at different stages.
